# Effect of Ionic Liquid on the Determination of Aromatic Amines as Contaminants in Hair Dyes by Liquid Chromatography Coupled to Electrochemical Detection

**DOI:** 10.3390/molecules17077961

**Published:** 2012-07-02

**Authors:** Thiago Mescoloto Lizier, Maria Valnice Boldrin Zanoni

**Affiliations:** Institute of Chemistry-State University “Julio de Mesquita Filho”-UNESP-Avenida Professor Francisco Degni, 55, Quitandinha, CEP: 14800-900, Araraquara-SP, Brazil; Email: tmlizier@yahoo.com.br

**Keywords:** ionic liquid in chromatography, BMIm[NTf_2_], HPLC with electrochemistry detection, hair dye, carcinogenic amines determination

## Abstract

The room temperature ionic liquid (IL) 1-butyl-3-methylimidazolium bis-(trifluorometanesulfonyl)imide BMIm[NTf_2_] was used as a novel medium for improvement of separation and quantization of 16 aromatic amines typically present as contaminants in consumer products and detected by HPLC coupled to an electrochemical detector. The aromatic amines, namely 4,4'-diaminodiphenylmethane, 4-chloroaniline, 2-methoxy-5-methyl-aniline, 3,3'-dimethylbenzidine, 2,4-diaminotoluidine, 2-chloro-4-nitroaniline, 4,4'-oxydianiline, aniline, 3,3'-dichlorobenzidine, benzidine, 4-aminobiphenyl, *o*-dianisidine, *o*-anisidine, *o*-toluidine, 4,4'-methylene-bis-2-chloroaniline and 2-naphthyl-amine are oxidized in methanol/BMIm[NTf_2_] at a potential around +0.68V to +0.93V *vs.* Ag/AgCl at a glassy carbon electrode, which is the base for their determination by HPLC/ED. Using the optimized conditions of methanol/BMIm[NTf_2_] 70:30 (v/v) as mobile phase, flow-rate of 0.8 mL·min^−1^, column CLC-ODS, E_ap_ = +1.0 V and T = 40 °C analytical curves were constructed for each of the tested amines. Good linearity was obtained in the concentration range of 1.09 mg·L^−1^ to 217 mg·L^−1^, with excellent correlation coefficients. The limits of detection reached 0.021 mg·L^−1^ to 0.246 mg·L^−1^ and good relative standard deviations (RSD, n = 3) were obtained from the measurements. Satisfactory recovery for each aromatic amine was achieved, ranging from 95 to 103%. The developed method was successfully applied to determine six aromatic amines present as contaminants in commercial hair dye samples.

## 1. Introduction

Aromatic amines are a class of chemicals essential in the plastic and chemical industries, and found as byproducts of the manufacture of compounds such as polyurethane foams, dyes, pesticides, pharmaceuticals and semiconductors. Aromatic amines can enter the aqueous environment as precursors from the synthesis of these compounds, via azo dye and nitroaromatic compound reductions [1,2], diesel exhaust, combustion of wood chips and rubber, tobacco smoke and grilled meats and fish and have been identified as potential carcinogens [3]. Since they have been designated as high priority pollutants, their presence in the environment must to be monitored at concentration levels lower than 30 mg·L^−1^ compatible with the limits allowed by the regulations. Epidemiological evidence on the relation between aromatic amines and cancer risk has been reviewed [4]. In addition, the prohibition on the use of certain azo dyes that release one of the 22 aromatic amines as provided in EU Regulation (EC) 1907/2006 on the Registration, Evaluation and Authorization of Chemicals (REACH), is directly applicable in all EU Member States. The main amines that are of great concern are: 4-aminobiphenyl, benzidine [4,4'-diaminobiphenyl], 4-chloro-*o*-toluidine [4-chloro-2-methylaniline], 2-napthylamine [2-aminonaphthalene], 4-chloroaniline, 4,4'-diaminodiphenylmethane [bis(4-aminophenyl)methane], 3,3'-dichlorobenzidine [4-(4-amino-3-chlorophenyl)-2-chloroaniline], 3,3'-dimethylbenzidine [4-(4-amino-3-methoxyphenyl)-2-methoxyaniline], *o*-dianisidine, 4,4'-methylene-bis-(2-chloroaniline) [4-[(4-amino-3-chlorophenyl)methyl]-2-chloroaniline], 4,4'-oxydianiline, *o*-toluidine [2-methylaniline], *o*-anisidine [2-methoxyaniline], 2-methoxy-5-methylaniline, 2,4-diaminotoluidine [2,4-diaminotoluene], 2-chloro-4-nitroaniline, aniline [phenylamine]. Therefore, several methods are proposed in literature for analysis of aromatic amines.

In general, the amines condemned by the IARC are limited to ppb levels in natural waters, which demands sensitive analytical methods such as those based on electrochemical oxidation, spectrophotometric analysis after derivatization reactions, thin layer chromatography, gas chromatography, liquid chromatography with various types of detectors and capillary electrophoresis [5,6,7,8,9,10,11,12,13,14].

The literature also describes examples for the determination of aromatic amines in environmental samples [15,16,17,18], foods [19,20], biological fluids [21,22,23] and from treatments leading to partial degradation of azo dyes [24,25,26,27,28,29]. As a disadvantage, they have high detection limits and need long pre-concentration processes for a good sensitivity, and derivatization processes to enhance the volatility of these amines when analyzed by gas chromatography, for instance. The high basicity, reactivity and polar nature of aromatic amines are responsible for different problems involved on the extraction and detection by chromatographic analysis [1]. The coupling of electrochemical detectors (ED) in High Performance Liquid Chromatography (HPLC) systems has shown great potential in the quantification of trace organic compounds in various matrices [30]. The method presents higher sensitivity than other conventional methods and raised some problems such as lack of baseline stabilization and adsorption of oxidized species from substrate or impurities onto the electrode surface. The separation of amine compounds at HPLC-ED condition still remains problematic due to the interactions with silanol groups in the chromatography columns. 

The special characteristics of room temperature ionic liquids (IL) for electrochemical applications have been the focus of several researchers [31,32,33]. They are robust compounds that present good conductivity, exhibit wide electrochemical potential windows [29,30] and, as materials composed of cations and anions, are able to solvate a large variety of organic and inorganic compounds, either polar or non-polar. In addition, some authors have used ionic liquids as mobile phase additives to improve the detection and separation of amines by liquid chromatography [34,35]. Usually, ionic liquids can compete for the silanol groups of stationary phase with the basic groups of the analytes and no polar alkyl groups of the stationary phase can interact with different alkyl groups of the heterocyclic ring or quaternary cation of the IL [36]. The ion-paring with cationic solutes [37] could efficiently shield the residual silanols, which should improve the peak shapes while reducing the chromatographic retention times of the basic analytes [38]. The high conductivity of some ionic liquids also removes the need for other supporting electrolytes and allow for a higher potential window necessary to oxidize the aromatic amines [39,40,41,42,43]. Martin-Calero [44] showed the beneficial effects of several ionic liquids (ILs) as mobile phase additives in high-performance liquid chromatography with electrochemical detection for determination of heterocyclic aromatic amines (HAs) in food samples. 

The aim of the present work was to develop an sensitive analytical method for the determination of 16 aromatic amines condemned by IARC and found in commercial hair dyes using high liquid chromatography coupled to an electrochemical detector by using the ionic liquid 1-butyl-3-methylimidazolium bis(trifluoromethanesulfonyl)imide (BMIm[NTf_2_]) in the mobile phase. 

## 2. Results and Discussion

### 2.1. Cyclic Voltammetric Investigations

**Figure 1:**
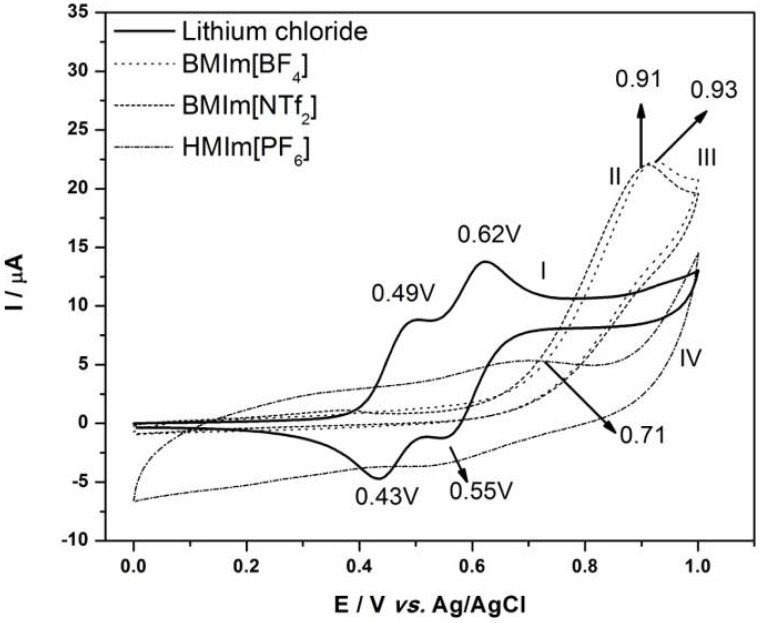
Cyclic voltammogram obtained for oxidation of 244 mg·L^−1^ of *o*-dianisidine in 4.2 g·L^−1^ of LiCl (I); *o*-dianisidine in 12.6 mg·L^−1^ of BMIm[NTf_2_] (II); *o*-dianisidine in 6.78 mg·L^−1^ BMIm[BF_4_] (III); *o*-dianisidine in 9.36 mg L^−1^ HMIm[PF_6_] (IV) at a glassy carbon electrode. Scan speed (ν) = 50 mV·s^−1^.

In order to optimize the experimental conditions required in the development of an electrochemical detection system previously the voltammetric oxidation of amines of interest on glassy carbon electrode in a methanol/ionic liquid medium was investigated. Cyclic voltammograms were recorded for each one of the 16 aromatic amines. Figure 1 shows the cyclic voltammograms obtained for oxidation of 244 mg·L^−1^*o*-dianisidine in 50:50 methanol/ionic liquid where the ionic liquid was 1-butyl-3-methylimidazolium bis(trifluoromethylsulfonyl)imide (BMIm[NTf_2_]), 1-butyl-3-methyl-imidazolium tetrafluoroborate (BMIm[BF_4_]) or 1-hexyl-3-methylimidazolium hexafluorophosphate (HMIm[PF_6_]). The voltammetric behaviors of the chosen model compound were similar in all cases. The oxidation in BMIm-BF_4_ presents one peak at 0.93V, attributed to the oxidation of the amine. In HMIm[PF_6_] the *o*-dianisidine is also oxidized in one step at 0.71 V and in BMIm[NTf_2_] its oxidation occurs at +0.91 V, respectively. This electrochemical behavior is slightly different in relation to the supporting electrolyte methanol/LiCl (4.2 g·L^−1^) where two anodic peaks at +0.49 V and +0.62 V and two cathodic peaks at +0.55 V and +0.43 V are observed, indicating that the amine group is probably oxidized in two steps of two electron transfer [45]. 

In IL the first peak is much smaller than the main peak around 0.66 V to 0.72 V, but in the LiCl it is inexistent. This behavior indicates that in HMIm[PF_6_] ionic liquid *o*-dianisidine probably forms an ionic pair with the anion of the IL, leading to formation of a pre-peak at less positive potential where the amine group is oxidized, however from BMIm[BF_4_] and BMIm[NTf_2_] ionic liquid no ionic pair forms, leading to formation of a more positive peak from the oxidation of the amine group. Taking into consideration the form and position of the peaks in the voltammogram methanol/BMIm[NTf_2_] was chosen the as the best supporting electrolyte for further studies.

**Figure 2:**
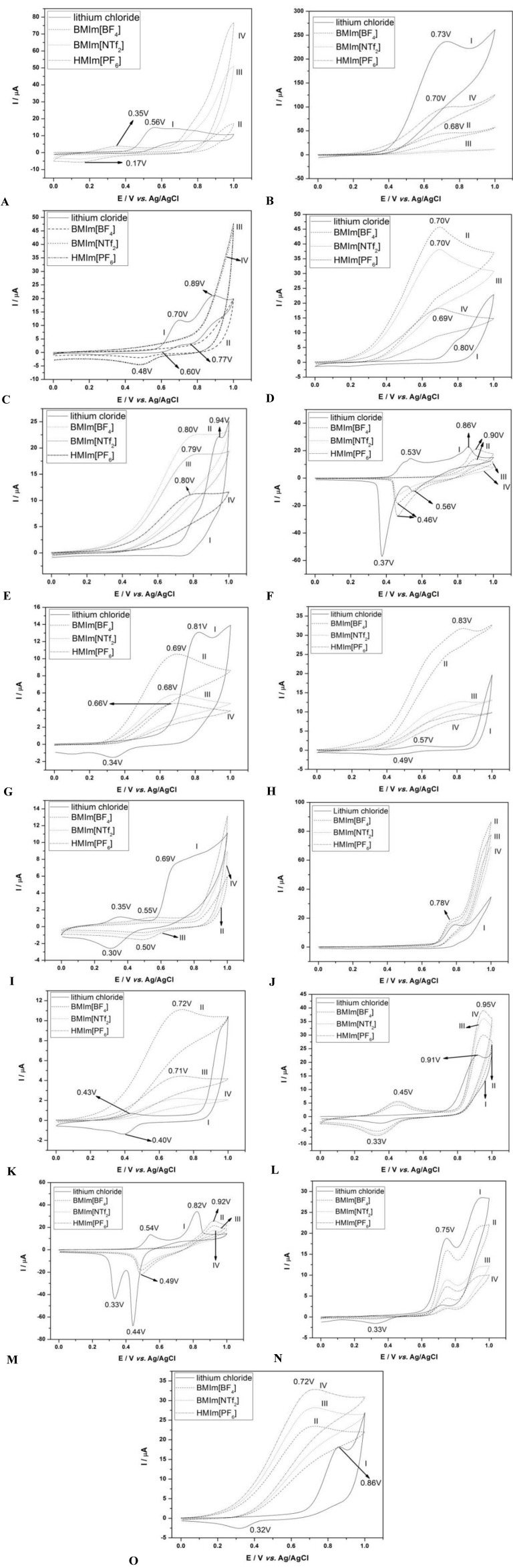
Cyclic voltammogram obtained for oxidation of 244 mg·L^−1^ of amine in the ionic liquid BMIm[NTf_2_] at a glassy carbon electrode. Scan speed: 50 mV·s^−1^. (**A**) 2,4-diaminotoluidine; (**B**) 2-chloro-4-nitroaniline; (**C**) 2-methoxy-5-methylaniline; (**D**) 2-naphtylamine; (**E**) 3,3'-dichlorobenzidine; (**F**) 3,3'-dimethylbenzidine; (**G**) 4,4'-diamino-diphenylmethane; (**H**) 4,4'-methylene-bis-chloroaniline; (**I**) 4,4'-oxydianiline; (**J**) 4-amino-biphenyl; (**K**) 4-chloroaniline; (**L**) aniline; (**M**) benzidine; (**N**) *o*-anisidine; (**O**) *o*-toluidine in (I) amine + methanol/0.1 mol·L^−1^ of Lithium chloride; (II) amine + methanol/BMIm[BF_4_] (50:50 v/v); (III) amine + methanol/BMIm[NTf_2_] (50:50 v/v); (IV) amine + methanol/HMIm[PF_6_] (50:50 v/v) at glassy carbon electrode. Scan rate = 50 mV·s^−1^.

The cyclic voltammograms obtained for 244 mg·L^−1^ of aromatic amines in methanol/BMIm[NTf_2_] (50:50) are shown in Figure 2. It is possible to observe the occurrence of two oxidation steps for the following amines: benzidine, 3,3'-dimethylbenzidine, 4,4'-oxydianiline, 4-chloroaniline and aniline. In contrast, only one oxidation step is observed for: *o*-anisidine, *o*-toluidine, 4,4'-methylene-bis-chloroaniline, 2-napthylamine, 4,4'-diaminodiphenylmethane, 2-methoxy-5-methylaniline, 2,4-diaminotoluidine, 2-chloro-4-nitroaniline, 4-aminobiphenyl, with no peak in the cathodic scan. A linear relationship is obtained for plots of peak currents (II_c_) *vs.* square-root scan rate.

The Table 1 shows the potential and peak currents from the anodic scan. This behavior indicates that all anodic processes are diffusion controlled and could be used to detect the aromatic amines by HPLC/ED.

**Table 1:** Voltammetric parameters obtained for oxidation of aromatic amines at glassy carbon electrode at 50 mV·s^−1^ in methanol/lithium cloride and methanol/BMIm[NTf_2_].

Amine compounds	LiCl				BMIm[NTf_2_]			
	E_a 1_ (V)	E_a 2_ (V)	Ip_a__I_ (µA)	Ip_a__II_ (µA)	E_a 1_ (V)	E_a 2_ (V)	Ip_a__I_ (µA)	Ip_c__II_ (µA)
**2,4-Diaminotoluidine**	+0.56	--	15.6	--	--	0.90	--	--
**2-Chloro-4-nitroaniline**	+0.73	--	12.8	--	+0.68	--	18.6	--
**2-Methoxy-5-methylaniline**	+0.70	+0.89	10.3	8.9	--	0.91	--	--
**2-Naphthylamine**	+0.80	--	14.5	--	+0.70	--	16.5	--
**3,3** **'-Dimethylbenzidine**	+0.53	+0.86	11.4	12.3	+0.46	+0.90	12.3	10.4
**4,4'-Diaminodiphenylmethane**	+0.81	-	13.5	--	+0.68	--	16.8	--
**4,4** **'-Methylene-bis-chloroaniline**	+0.57	-	9.3		--	+0.85	--	--
**4,4** **'-Oxydianiline**	+0.35	+0.69	6.7	6.2	+0.55	+0.50	6.2	6.8
**4-Aminobiphenyl**	+0.78	--	9.8	--	+0.78	--	9.1	--
**4-Chloroaniline**	+0.43	--	8.6		+0.71	--	12.8	--
**Aniline**	+0.91	--	9.6	--	+0.45	0.95	10.3	14.4
**Benzidine**	+0.54	+0.82	10.3	13.2	+0.92	--	12.4	
***o*-Anisidine**	+0.75	--	9.1	--	+0.75	--	10.2	--
***o*-Dianisidine**	+0.49	+0.62	14.4	14.8	+0.93	--	15.2	--
***o*-Toluidine**	+0.86	--	12.8	--	+0.72	--	14.2	--

These results indicate that all the aromatic amines investigated presented an amine group oxidized at a potential from 0.45 V to 0.93 V, which could be the basis for its selective amperometric detection when coupled to HPLC.

### 2.2. HPLC/ED Optimizations

#### 2.2.1. Optimization of Mobile Phases

The development of an analytical method for aromatic amine determination based on HPLC/ED was first investigated testing the parameters concentration of solvent and concentration of ionic liquid in the mobile phase, flow rate, applied potential and type of solvent. The composition and concentration of the supporting electrolyte are known to have a marked influence on the electrochemical detection response [44]. For this, several supporting electrolytes using methanol/water + 0.1% formic acid and Acetonitrile/water + 0.1% of formic acid varied from 60:40 (v/v); 70:30 (v/v); 80:20 (v/v) and 85:15 (v/v). Using some chromatographic parameters such as retention time, resolution, it was concluded that the best conditions for separation of the investigated compounds were obtained for methanol/water + 0.1% of formic acid 70:30 (v/v). In order to improve the peak resolution in the chromatograms of aromatic amines, the influence of mobile phase flow-rate through the column was investigated comparing chromatograms recorded from 0.6 to 1.2 mL·min^−1^. The flow rate can be an important parameter to optimize the analysis, since the flow-rate variations lead to significant changes in the column efficiency, thereby affecting the number of theoretical plates (N) in the column. The results obtained indicate that an upper limit of flow-rate of 1.2 mL·min^−1^ can be used, since higher flow-rates cause excessive column pressure usually limited to 350 Kgf. Flow-rates lower than 0.6 mL·min^−1^ result in separation times longer than 40 min, which were considered unacceptable for this methodology. In order to avoid these anomalies, the chromatograms were recorded using a careful polish of the working electrode surface before each increase in the flow-rate, through a clean electrochemistry program set changing from +1.0 V to −1.0 V during 10 s. Using these experimental conditions, a flow-rate of 0.80 mL·min^−1^ was considered as the optimum value for the resolutions in the chromatogram with excellent repeatability.

An electrochemical detector requires the application of a potential high enough to oxidize all the aromatic amines give a high response, but not so high as to compete with the supporting electrolyte discharge. Thus, taking into consideration the voltammograms obtained under static conditions the detector was configured to work in amperometric mode from +0.50 V to +1.2 V. For this, curves of current *vs.* potential were constructed for each amine (24 mg·L^−1^) in methanol/LiCl (4.23 g·L^−1^) in proportion 70:30 v/v as mobile phase, at a flow-rate of 0.80 mL·min^−1^. 

**Figure 3:**
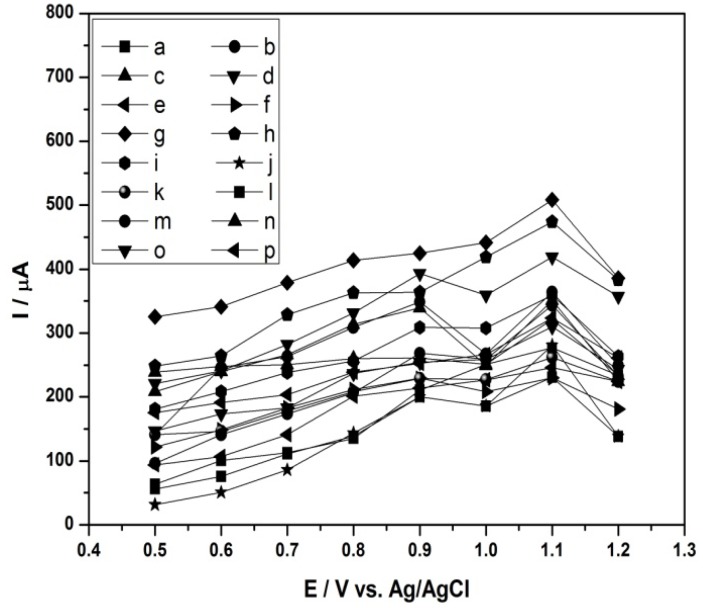
Hydrodynamic voltammograms of a 24 mg·L^−1^ standard of **a**: 4,4'-oxydianiline; **b**: aniline; **c**: 2,4-diaminotoluidine; **d**: *o*-dianisidine; **e**: benzidine; **f**: 4,4'-methylene-bis-(2-chloroaniline); **g**: 2-naphthylamine; **h**: *o*-toluidine; **i**: *o*-anisidine; **j**: 4,4'-diamino-diphenylmethane; **k**: 3,3'-dimethylbenzidine; **l**: 2-chloro-4-nitroaniline; **m**: 4-amino-biphenyl; **n**: 2-methoxy-5-methylaniline; **o**: 4-chloroaniline; **p**: 3,3'-dichlorobenzidine solution at HPLC coupled to an electrochemical detector (glassy carbon electrode). Mobile phase: methanol/water 70:30 (v/v) + 0.1% formic acid; Flow-rate: 0.8 mL·min^−1^; Column: Shimpack (Shimadzu) CLC-ODS; T: 40 °C.

Figure 3 shows the current curves obtained as a function of applied potential for the investigated aromatic amines. The analysis of Figure 3 indicates that higher peak intensities are observed for applied potentials (E_o_) from 1.0 V. Therefore, an applied potential value of +1.0 V was chosen as optimum to detect all amines.

**Figure 4:**
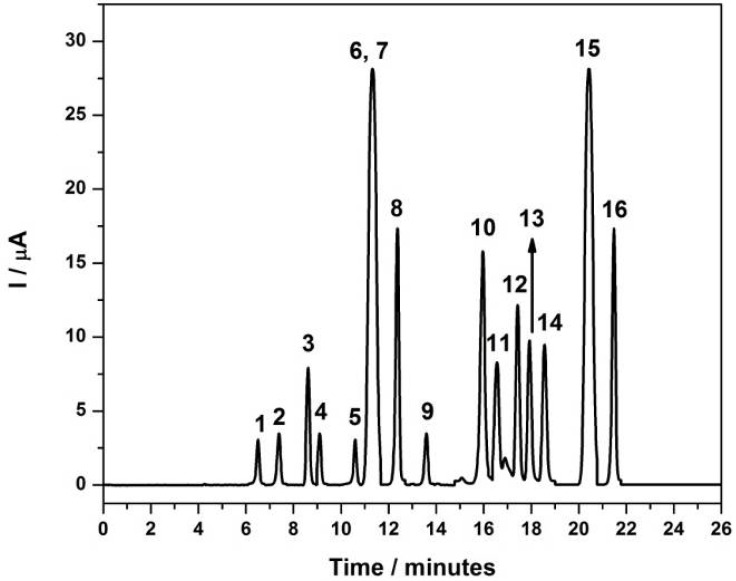
HPLC/ED chromatograms obtained for 20 μL of standard solution of 24 mg·L^−1^ of the selected amines.Mobile phase:MeOH/water + 0.1% formic acid 70:30 (v/v) T= 40°C, flow = 0.80 mL·min^−1^; C_18_ phase column, E = +1.00 V. **1**: 4,4'-diaminodiphenyl-methane; **2**: 4-chloroaniline; **3**: 2-methoxy-5-methylaniline; **4**: 3,3'-dimethylbenzidine; **5**: 2,4-diamintoluidine; **6**: 2-chloro-4-nitroaniline; **7**: 4,4'-oxydianiline; **8**: aniline; **9**: 3,3'-dichlorobenzidine; **10**: benzidine; **11**: 4-aminobiphenyl; **12**: *o*-dianisidine; **13**: *o*-anisidine; **14**: *o*-toluidine; **15**: 4,4'-methylene-bis-2-chloroaniline; **16**: 2-naphthylamine.

Figure 4 shows a typical chromatogram for standard solutions of 24 mg·L^−1^ of amine in methanol/LiCl (4.23 g·L^−1^) in proportion 70:30 v/v as mobile phase and a flow-rate of 0.80 mL·min^−1^, E_oxid_ = +1.0 V for each aromatic amines. The elution of the examined compounds was completed in a time of 25 min during a chromatographic run. Peak identifications were based on their retention times, which were confirmed comparing with standard samples of the aromatic amines and gave the following sequence: 4,4'-diaminodiphenylmethane (6.48 min); 4-chloroaniline (7.42 min); 2-methoxy-5-methylaniline (8.61 min); 3,3'-dimethylbenzidine (9.09 min); 2,4-diaminotoluidine (10.6 min); 2-chloro-4-nitroaniline (11.30 min); 4,4'-oxydianiline (11.30 min); aniline (12.43 min); 3,3'-dichloro-benzidine (13.60 min); benzidine (15.97 min); 4-aminobiphenyl (16.53 min); *o*-dianisidine (17.40 min); *o*-anisidine (17.95 min); *o*-toluidine (18.59 min); 4,4'-methylene-bis-2-chloroaniline (20.41 min); 2-naphtylamine (21.44 min). From this chromatogram is possible to observe that no separation of the peaks corresponding to 2-chloro-4-nitroaniline (11.30 min) and 4,4'-oxydianiline (11.30 min) was obtained. In addition, there is peak broadening, leading to poorer analytical performance.

Theoretically, the conversion efficiency of a thin-layer amperometric cell, as used in this study, could be improved by decreasing the flow rate through the cell, thereby increasing the residence time of the analyte in contact with the working electrode, but all the experiments show unsatisfactory results. 

#### 2.2.2. Separation of Aromatic Amines After Addition of BMIm[NTf2] in the Mobile Phase

**Figure 5:**
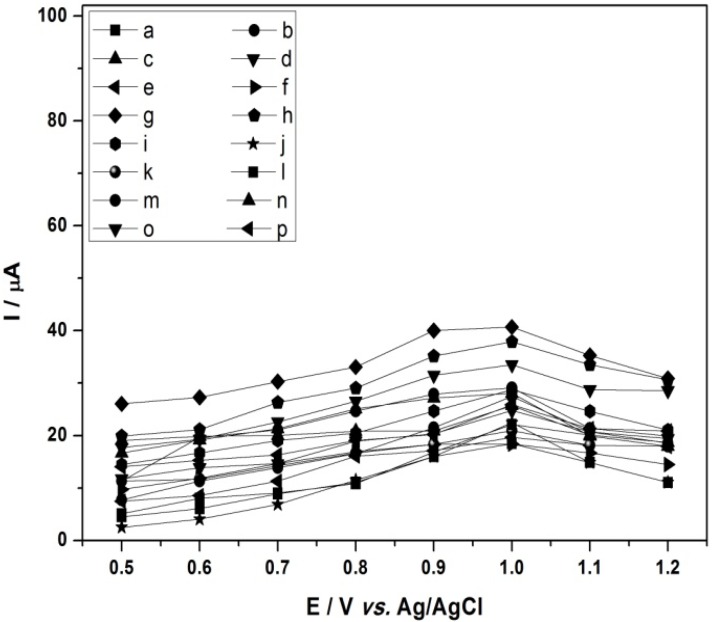
Hydrodynamic voltammograms of a 24 mg·L^−1^ standard of: **a**: 4,4'-oxydianiline; **b**: aniline; **c**: 2,4-diaminotoluidine; **d**: *o*-dianisidine; **e**: benzidine; **f**: 4,4'-methylene-bis-(2-chloroaniline); **g**: 2-naphthylamine; **h**: *o*-toluidine; **i**: *o*-anisidine; **j**: 4,4'-diaminodiphenyl-methane; **k**: 3,3'-dimethylbenzidine; **l**: 2-chloro-4-nitroaniline; **m**: 4-aminobiphenyl; **n**: 2-methoxy-5-methylaniline; **o**: 4-chloroaniline; **p**: 3,3'-dichlorobenzidine solution at HPLC coupled to an electrochemical detector (glassy carbon electrode). Mobile phase: methanol/water 70:30 (v/v) +12.6 mg·L^−1^ BMIm [NTf_2_]. Flow-rate: 0.8 mL·min^−1^. Column: Shimpack (Shimadzu) CLC-ODS; T: 40 °C.

With the aim to increase the resolution of these peaks further studies were carried out on solutions of the 16 aromatic amines in methanol/water in proportion 70:30 + 12.6 mg·L^−1^ BMIm[NTf_2_]. The concentration of the ionic liquid constituent is known to have a marked influence on the electrochemical detection response [46]. For this, cyclic voltammograms were recorded comparing three ionic liquids in methanol/water varying the concentration from 4.22 mg·L^−1^ to 12.6 mg·L^−1^. The ionic liquid HMIm[PF_6_] did not show sufficient solubility in water. The ionic liquid BMIm[BF_4_] showed no significant effect on the resolution and peak height. Thus, the ionic liquid BMIm[NTf_2_] presented best voltammograms, in addition better curves were obtained using 12.6 mg·L^−1^. Hydrodynamic voltammograms constructed using current *vs.* potential for each amine (24 mg·L^−1^) in methanol/water + 12.6 mg·L^−1^ BMIm[NTf_2_] in proportion 70:30 v/v as mobile phase, and a flow-rate of 0.80 mL·min^−1^ are shown in Figure 5.

Figure 5 shows the obtained curves of current in function of applied potential for the investigated aromatic amines. It indicates that higher peak intensities are observed for applied potentials (E_o_) from 0.90 to 1.0 V. Therefore, an applied potential value of +1.0 V was chosen the optimum one which can promote the most selective response and high sensitivity to detect all amines using the proposed method.

**Figure 6:**
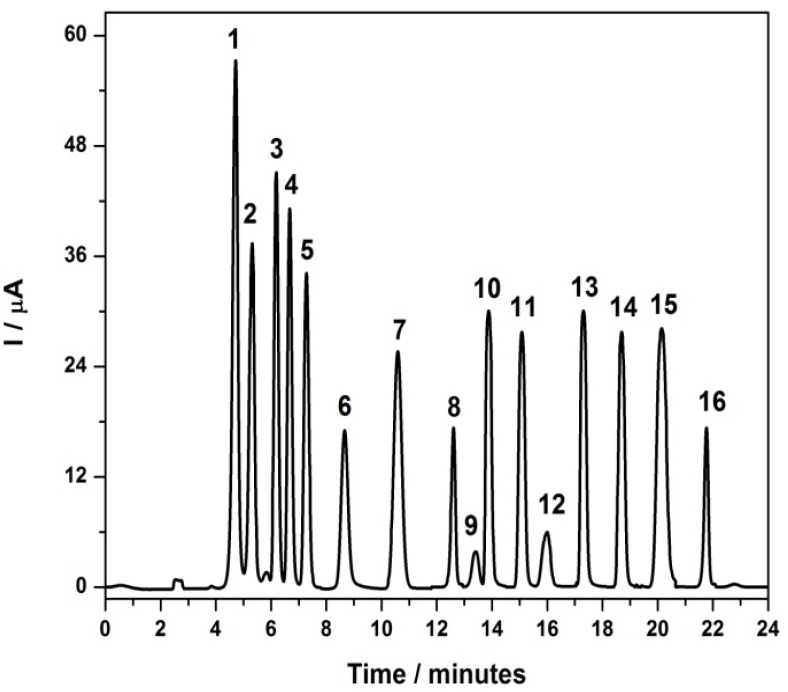
HPLC/ED chromatograms obtained for 20 mL of standard solution of 24 mg·L^−1^ of the selected amines. Mobile phase: MeOH/water +30 mM BMIm[NTf_2_] 70:30 (v/v); T: 40 °C, flow-rate: 0.80 mL·min^−1^; C_18_ phase column, E: +1.00 V. **1**: 4,4'-diaminodiphenyl-methane; **2**: 3,3'-dimethylbenzidine; **3**: 2-methoxy-5-methylaniline; **4**: 4-chloroaniline; **5**: 2,4-diaminotoluidine; **6**: 2-chloro-4-nitroaniline; **7**: 4,4'-oxydianiline; **8**: 4-aminobiphenyl; **9**: 3,3'-dichlorobenzidine; **10**: benzidine; **11**: aniline; **12**: *o*-dianisidine; **13**: *o*-anisidine; **14**: *o*-toluidine; **15**: 2-naphthylamine; **16**: 4,4'-methylene-bis-2-chloroaniline.

A typical chromatogram for a mixture of standard solutions containing 24 mg·L^−1^ of aromatic amines of electrochemical detection using the best conditions, previously: flow-rate of 0.8 mL·min^−1^, methanol/water + 30 mmol·L^−1^ BMIm[NTf_2_]; (70:30 v/v), E_ox_ = +1.0 V is shown in Figure 6. 

The retention times were found to become slightly different in the presence of liquid ionic, following the sequence: 4,4'-diaminodiphenylmethane (4.7 min); 3,3'-dimethylbenzidine (5.3 min); 2-methoxy-5-methylaniline (6.1 min); 4-chloroaniline (6.7 min); 2,4-diaminotoluidine (7.2 min); 2-chloro-4-nitroaniline (8.6 min); 4,4'-oxydianiline (10.6 min); 4-aminobiphenyl (12.6 min); 3,3'-dichlorobenzidine (13.4 min); benzidine (13.8 min); aniline (15.1 min); *o*-dianisidine (15.9 min); *o*-anisidine (17.3 min); *o*-toluidine (18.7 min); 2-naphthylamine (20.1 min); 4,4'-methylene-bis-2-chloroaniline (21.7 min). The elution of the examined compounds was completed in a time of 22 min during a chromatographic run. Peak identifications were based on their retention times, which were compared with the peaks from chromatograms of the standard solutions of all aromatic amines investigated.

The results indicated that the addition of the soluble liquid ionic BMIm[NTf_2_] in water gives a great improvement in the resolution, peak height and shape of the chromatographic peaks since these are dependent on the kinetic interaction between the silanol groups of the stationary phase of the column and the analytes. The vast majority of the aromatic amines are positively charged under the experimental conditions, which causes strong interactions with the residual silanols of the chromatographic column that are negatively charged. The addition of ionic liquid in the mobile phase causes a blocking of the silanols interaction reducing the peak broadening. The ionic liquid cations are mostly responsible for these interactions with the stationary phase silanol groups. In addition, the cations of the IL can form ionic pairs with the aromatic amines and decrease their adsorption on the electrode surface which usually disturbs the baseline. These combined effects influence the separation mechanisms occurring on the stationary phase, decreases the retention times of the amines and alters the elution order when comparing with the analysis performed in the absence of the ionic liquid. 

The results thus indicate that ionic liquids can play an important role in the analysis of aromatic amines by HPLC/ED. The strong proton-acceptor properties of these IL can be utilized to suppress the deleterious effects of free silanols on liquid chromatographic separations. The chaotropic character of the anion may introduce ion-pairing with cationic solutes and adsorption on the stationary phase. The hydrophobicity of the cation may further induce stationary phase adsorption. All these factors in combination offer a simple way to detect aromatic amines at low level.

#### 2.2.3. Analytical Curves

The analytical HPLC/ED curves based on the relation of peak area and concentration were preferred instead of peak height for all the 16 aromatic amines in order to eliminate the possible effects of species adsorbed on the electrode surface, which could prejudice the quantitative response. The analytical curves for each aromatic amine were constructed by plotting the peak area against the concentration and a linear range was obtained from 1.09 mg·L^−1^ to 217 mg·L^−1^ (Table 2). 

**Table 2:** Analytical parameters HPLC/ED from 16 aromatic amines, flow rate: 0.8 mL·min^−1^, mobile phase: methanol/water +12.64 mg·L^−1^ of BMIm[NTf_2_], E = +1.0 V.

Amines	A **	B ***	N	R	SD	L.O.D.	L.O.Q.	As		Np		Rec.
								1	2	1	2	
**2,4-Diaminotoluidine**	4350.89	2035.16	5	0.998	686.0	0.110	0.337	0.85	0.92	31211.1	9421.9	100
**4,4** **'-Oxydianiline**	16596.20	2424.22	6	0.993	795.6	0.108	0.328	--	0.90	6491.8	5842.1	98
**Benzidine**	24.50	70.04	6	0.997	26.13	0.123	0.374	0.84	0.96	24214.4	22548.7	97
**Aniline**	5286.40	1105.63	6	0.996	664.8	0.098	0.600	0.78	1.02	42642.3	26437.0	99
***o*-Dianisidine**	7178.19	1813.43	6	0.997	597.8	0.303	0.113	0.80	0.94	190096.0	31604.9	100
***o-*Anisidine**	−8505.88	2230.6	6	0.998	680.3	0.103	0.303	0.77	0.96	89301.4	56808.3	103
***o*-Toluidine**	26373.5	2049.62	6	0.997	651.4	0.091	0.276	0.82	0.95	95378.0	66528.4	100
**2-Naphthylamine**	48572.45	4245.71	6	0.999	128.7	0.101	0.305	0.78	0.98	127925.4	45033.3	103
4,4'-Methylene-bis-2-chloroaniline	4549.79	1818.46	6	0.998	695.8	0.126	0.382	0.81	0.97	28985.1	156528.1	100
**4,4'-Diaminodiphenyl-methane**	181.89	50.69	9	0.999	21.2	0.117	0.416	0.79	0.96	11844.7	1972.8	99
**3,3** **'-Dimethylbenzidine**	−753.02	250.09	9	0.997	162.5	0.021	0.638	0.78	0.99	22901.8	4956.2	95
**2-Methoxy-5-methyl-aniline**	317.02	81.64	9	0.999	88.5	0.036	1.078	0.78	0.95	20544.4	6833.8	100
**4-Chloroaniline**	−108.03	72.00	9	0.997	26.6	0.120	0.368	0.76	0.98	39151.2	7932.9	105
**2-Chloro-4-nitroaniline**	−342.46	196.29	9	0.999	82.8	0.163	0.495	--	0.96	6491.8	9216.7	98
**4-Aminobiphenyl**	433.67	166.55	9	0.999	82.9	0.162	0.497	0.79	0.98	42642.2	52399.4	97
**3,3** **'-Dichlorobenzidine**	−339.63	464.56	9	0.999	120.6	0.246	2.021	0.81	0.96	51680.4	34110.3	96

L.O.D.: detection limited (mg·L^−1^); L.O.Q.: quantification limited (mg·L^−1^). ** linear coefficient; *** slope; # standard deviation; N: number of measures; R: correlation coefficient; As: symmetric (1: without IL; 2:with IL); Np: number of theory plates (1: without IL; 2:with IL) ; Rec.: Recovery.

### 2.3. Evaluation of Aromatic Amines in Commercial Dyeing Sample

The accuracy and recovery of the method were determined by spiking water samples with aromatic amine standards at a concentration level of 24 mg·L^−1^, whereby the samples were filtered through Millipore MILLEX filters and injected immediately. Extractions and analyses were performed in triplicate. The results obtained by the standard addition method or comparing calibration curves are shown in Table 1. Recoveries ranging from 95 to 105% of aromatic amines were obtained using the proposed method. This is a good evidence of the accuracy of the proposed method. 

**Figure 7:**
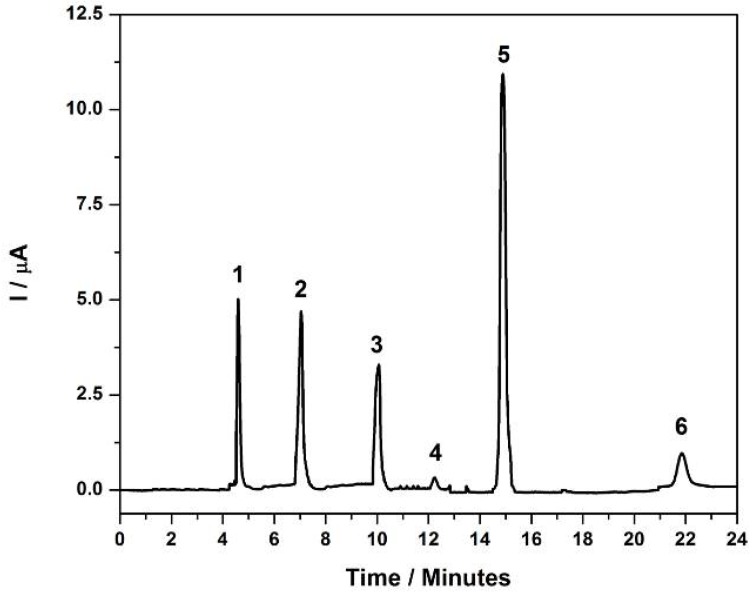
HPLC/ED chromatograms obtained for 20 μL of commercial solution of hair dye sample. Mobile phase: MeOH/water + 30 mM BMIm[NTf_2_] 70:30 (v/v) T: 40 °C, flow-rate: 0.80 mL·min^−1^; C_18_ phase column, E = +1.00 V. **1**: 4,4-diaminodiphenylmetame; **2**: 2,4-diaminotoluidine; **3**: 4,4'-oxydianiline; **4**: 4-aminobiphenyl; **5**: aniline; **6**: 4,4'-methylene-bis-2-chloroaniline.

The direct analysis of the aromatic amines in a commercial samples of hair dyes was not possible, since there is great interference of compounds in the sample in the background signal, but the chromatograms obtained under optimized condition after sample clean-up (Section 3.2.3) showed good performance and the HPLC/ED can be more sensitive. The method was applied to commercial dyes and indicated the occurrence of six amines (Figure 7), identified as 4,4'-diaminodiphenylmethane (5.21 mg·L^−1^), 2,4-diaminotoluidine (4.32 mg·L^−1^), 4,4'-oxydianiline (39.6 mg·L^−1^), 4-aminobiphenyl (5.07 mg·L^−1^), aniline (9.95 mg·L^−1^) and 4,4'-methylene-bis-2-chloroaniline (4.567 mg·L^−1^). It may be noted that various amines were found in the product formulation in the commercial dyes, but only 4,4'-oxydianiline was present in a higher concentration than permitted. This shows the danger that users and especially beauty professionals and cosmetologists run in contact with these compounds because they can cause cancer and other health problems. 

## 3. Experimental

### 3.1. Reagents

The studied amines were 2-naphthylamine (98%), 4,4'-methylene-bis-(2-chloroaniline) (85%), aniline (99%), *o*-toluidine (98%), 4,4'-oxidianiline (98%), *o*-dianisidine (98%), *o*-anisidine (99%), 3,3'-dimethylbenzidine (97%), 2-methoxy-5-methylaniline (99%) and 4-aminobiphenyl (90%) purchased from Sigma-Aldrich. 2,4-diaminotoluidine (98%), benzidine (98%), 4,4'-diamino-bis-phenylmethane (97%), 4-chloroaniline (99%) and 2-chloro-4-nitroaniline (98%) purchased from Fluka. 3,3'-Dichlorobenzidine (99%) purchased from Supelco. Stock standard solutions in methanol containing 50 mg·L^−1^ were prepared. These solutions were kept refrigerated at 0 °C and protected from light. Dilutions were made from stock solutions as needed. All samples were filtered through a Millex filter. The ionic liquids 1-butyl-3-methylimidazolium bis(trifluorometanesulfonyl)imide (BMIm[NTf_2_]), 1-butyl-3-methylimidazolium tetrafluoroborate (BMIm[BF_4_]), and 1-hexyl-3-methylimidazolium hexafluorophosphate (HMIm[PF_6_]), were purchased from Sigma-Aldrich. Acetonitrile and methanol were of HPLC-grade supplied by J.T. Baker. Water was purified using a Milli-Q system from Millipore. 

### 3.2. Instrumentation

#### 3.2.1. Voltammetric Analysis

Voltammetric measurements were performed using a PGSAT 30 potentiostat/galvanostat with a conventional three electrode arrangement. A glassy carbon electrode (2.5 mm diameter) was utilized as working electrode, the reference electrode was of Ag/AgCl and a Pt wire was the counter electrode. The glassy carbon electrode was polished with 1 μm alumina slurries on lapping pads. All the voltammograms were recorded after 5 s equilibration time and at a scan rate of 50 mV·s^−1^. The samples were prepared dissolving 244 mg·L^−1^ the aromatic amines in 10 mL methanol/LiCl 0.1 mol·L^−1^ or in 10 µL of methanol/ionic liquid that were mixed until completely homogeneous. All solutions were purged with high purity nitrogen for 10 min prior to recording the voltammograms, and a continuous stream of nitrogen was passed over the solutions during measurements. 

#### 3.2.2. HPLC Analysis

All the HPLC analysis coupled to an electrochemical detector was carried out using a Metrohm System 871 advanced Bioscan, with a 818 IC pump. Data acquisition and process were accomplished with an IC Net Workstation (Metrohm, Herisau, Switzerland). The separation was performed on a Phenomenex Luna C_18_ column (250 mm × 4.6 mm, 5 μm particle size) and a Shimadzu pre-column. A Metrohm Bioscan electrochemical cell was used, where the glassy carbon electrode is inserted as working electrode, a Variocell reference electrode with solid-phase Ag/AgCl and Pt as counter electrode. The aromatic amines were separated using methanol/ionic liquids or methanol/LiCl. All samples were filtered before each injection using Millex Millipore filters (0.45 μm). Ionic liquids are obtained from Sigma-Aldrich. 

#### 3.2.3. Analysis of Dyes in the Commercial Sample

The occurrence of aromatic amines was tested in a commercial hair dye sample assigned code HF 65, from ARIANOR. The following procedure was adopted: in a separatory funnel was added 3.00 mL of the commercial sample dissolved in 3.00 mL of dichloromethane (in triplicate). The organic phase rich in aromatic amine was evaporated and then dissolved in methanol. The samples were filtered through a 0.45 μm MILLEX filter and injected into the HPLC using the procedure described previously.

## 4. Conclusions

There is a strong consumer demand for hair dyes and for new analytical methods that identify and quantify these dyes. Our findings indicate that the use of room temperature ionic liquids as mobile-phase additives in high performance liquid chromatography (HPLC) offers great advantages in the separation of a wide variety of carcinogenic amines, especially using electrochemical detection. The method based on HPLC/ED has been shown here as being capable of determining very low levels of carcinogenic amines in hair dyes by using methanol/water + BMIm[NTf_2_] (70:30 v/v) as mobile phase. Even, at low concentrations in mobile phases, the anions and cations of the ionic liquid dramatically affect the retention times and resolution of the target compounds. Our findings provide a fast and simple method for the analysis of amines as contaminants in hair dye products using a very simple pre-treatment step. The proposed method is suggested as a good alternative for the routine quality control of this type of dyes in different matrices demanding quick and low cost analysis.
